# Ultrasound Guided Venous Access for Cardiac Devices: Defining Learning Curve for Safety, Efficacy, and Radiation Exposure

**DOI:** 10.1111/pace.70021

**Published:** 2025-07-31

**Authors:** Anish N Bhuva, Hnin Zaw, Adam Graham, Amal Muthumala, Philip Moore, Mehul Dhinoja

**Affiliations:** ^1^ Department of Cardiac Electrophysiology Barts Heart Centre, Barts Health NHS Trust London UK; ^2^ Institute of Cardiovascular Sciences University College London London UK

**Keywords:** axillary vein, learning curve, pacemaker, radiation, ultrasound

## Abstract

**Background:**

There is limited real‐world experience of the learning curve for ultrasound (US) guided axillary venous access for cardiac device implantation, and it is usually performed before cutaneous incision. We investigated the learning curve, radiation exposure, safety, and efficacy of US‐guided venous access in standard workflow.

**Methods:**

US‐guided access was performed by an experienced electrophysiologist with no prior application of the technique by using a standard vascular US probe and minimal modification to workflow. The learning curve was evaluated using access time (needle‐to‐wire time). Complications were recorded until hospital discharge, and efficacy was defined by procedural success. Radiation dose savings were estimated based on fluoroscopy time for access, and a control group underwent conventional fluoroscopy landmark‐guided access (*n* = 44 punctures).

**Results:**

147 US‐guided punctures were performed in 74 patients for one (8%), two (71%), or three (17%) leads, or upgrades (4%). Access was successful in 97% (*n* = 72). There were no access‐related peri‐procedural complications. First US‐guided access time was 30 seconds (interquartile range [IQR]: 17,60), and was similar to fluoroscopy‐guided access time (43 seconds, IQR: 24,58; *p* = 0.45). Access time stabilized after 45 procedures, decreasing from 81 (IQR: 61,90) to 16 seconds (IQR: 10,20) from the first to the last 15 procedures (*p* < 0.001).

96% (*n* = 69) did not require fluoroscopy. 4% (*n* = 3) required 1 second fluoroscopy to confirm wire position after difficult passage. Estimated radiation exposure saving from controls was 30 seconds (IQR: 15,78) of fluoroscopy, resulting in 0.4 (IQR: 0.26,1.7) mGy cumulative skin dose, equivalent to 1.3 (95% confidence interval: 0.26,1.45) patient chest radiograph radiation exposure.

**Conclusion:**

US‐guided axillary venous access for cardiac device implantation is feasible in a standard workflow and reduces radiation exposure. The learning curve time is acceptable, and the procedure is safe, even during training.

**Abbreviations**
BMI: Body mass index
ICDs: Implantable cardioverter‐defibrillators
IQR: Interquartile range
US: Ultrasound

## 1 Introduction

There is no single ideal technique for venous access during implantation of pacemakers and implantable cardioverter‐defibrillators (ICDs). Cephalic or extrathoracic subclavian vein puncture are the most common approach, but either has limitations [1, 2]. Fluoroscopy and venography guidance can improve safety and efficacy, but require radiation exposure to patient and operator, or ipsilateral peripheral cannulation and contrast administration. Direct visualization and depth perception using ultrasound (US) facilitate central venous cannulation, and so its use is recommended by professional bodies in the United States and Europe for patient safety [3, 4]. US guidance is not yet commonly used for cardiac device implantation [1], despite the first report of US‐guided axillary access over 20 years ago [5], and recent randomized trials demonstrating similar success rates to subclavian puncture or cephalic access [6, 7].

Changes to workflow and the learning curve represent barriers to more widespread adoption. The technique used in randomized trials requires changing conventional procedural steps so that venous puncture and guidewire insertion are performed prior to cutaneous incision [6, 7, 8]. The need to perform cutaneous incision around the site of wire entry requires significant adaptation by a new operator. There is limited real‐world experience [6, 9, 10, 11, 12, 13], but low success rates (69%–90%) have been reported, particularly in the training phase [5, 6, 12]. Maneuvers that facilitate the ease of the technique may cut the learning curve and encourage uptake by other operators.

Venous access following conventional implant steps, and concurrent infusion of intravenous fluids to identify the vein, may be helpful. We describe US‐guided axillary venous access adapted to standard implant workflow, including application to device upgrade procedures, and using a standard vascular US probe. We report procedural safety, efficacy, self‐taught learning curve, and estimate radiation dose‐saving.

## 2 Methods

### 2.1 Study Design and Population

This was an observational single‐center study. The study included consecutive patients undergoing cardiac device implantation by a single operator between June 2018 and November 2019. The implanter was an experienced electrophysiologist (>1000 procedures) with no prior application of the technique, but familiarity with US‐guided femoral venous access.

The study protocol conformed to the ethical guidelines of the 1975 Declaration of Helsinki. The study met criteria for operational improvement activity and was approved as a clinical effectiveness project by the Institutional Quality Improvement Review Board (ID 11345).

### 2.2 Procedural Preparation

Procedures were performed in the electrophysiology laboratory of a tertiary referral hospital. Anticoagulation continued uninterrupted for the procedure as per local protocol. Antibiotic prophylaxis was administered one hour before the procedure. A contrast venogram was performed prior to proceeding with device upgrades.

The patient underwent standard preparation by skin preparation and draping, so the area accessible contained the deltopectoral groove, and the clavicle formed the upper border.

**FIGURE 1:**
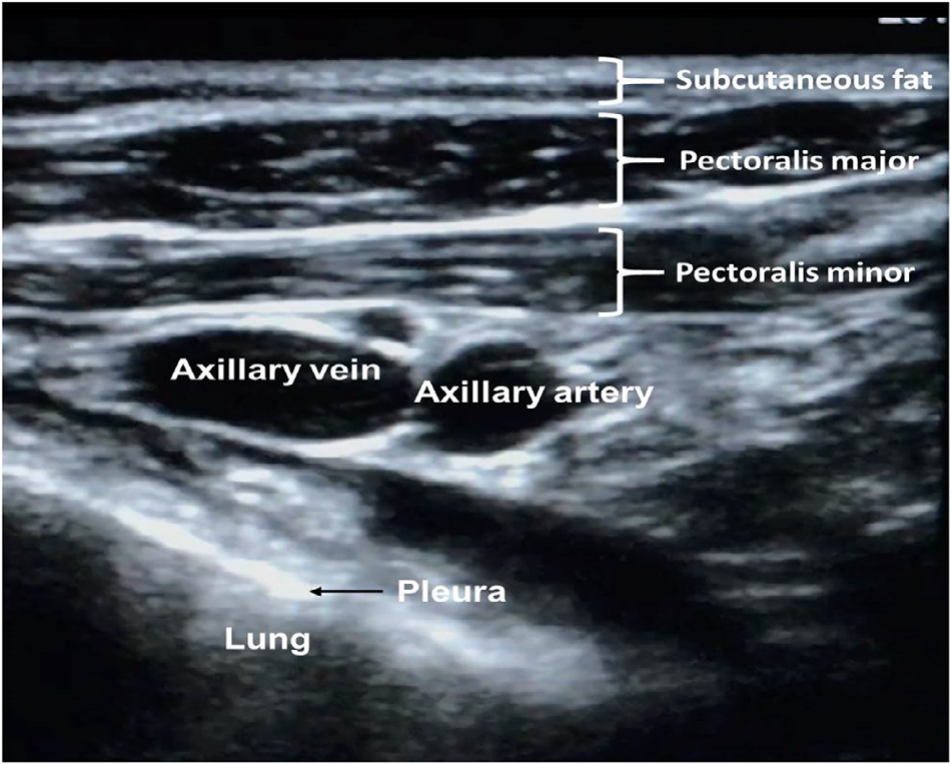
Ultrasound‐guided axillary vein anatomy. Ultrasound images are obtained by placing a vascular probe below and perpendicular to the clavicle. [Colour figure can be viewed at wileyonlinelibrary.com]

**FIGURE 2:**
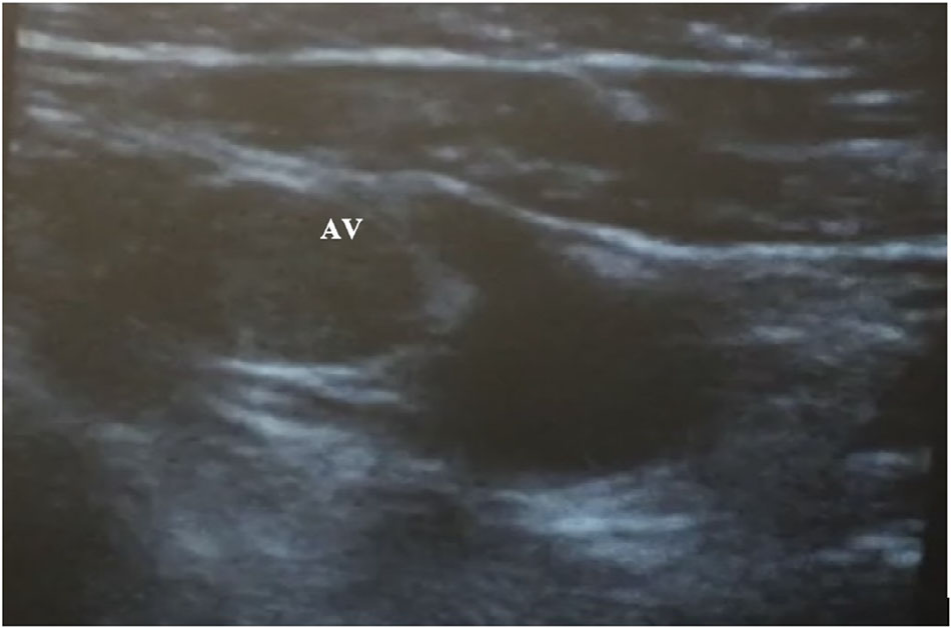
Axillary vein (AV) opacified with intravenous fluid. [Colour figure can be viewed at wileyonlinelibrary.com]

A standard vascular US probe (13‐6 MHz Sonosite vascular [Fujifilm Sonosite, Bothwell, WA, USA]) was prepared with US gel and covered by a long sterile sheath. It was placed in the deltopectoral groove and moved medially and upward toward the clavicle until the axillary vein and artery could be seen in the out‐of‐plane projection, with the vein in the middle of the imaging field (Figure 1). Intravenous fluids were continuously administered through an ipsilateral peripheral cannula. This provided bubble opacification and venous engorgement to facilitate detection and differentiation from the axillary artery (Figure 2).

**FIGURE 3:**
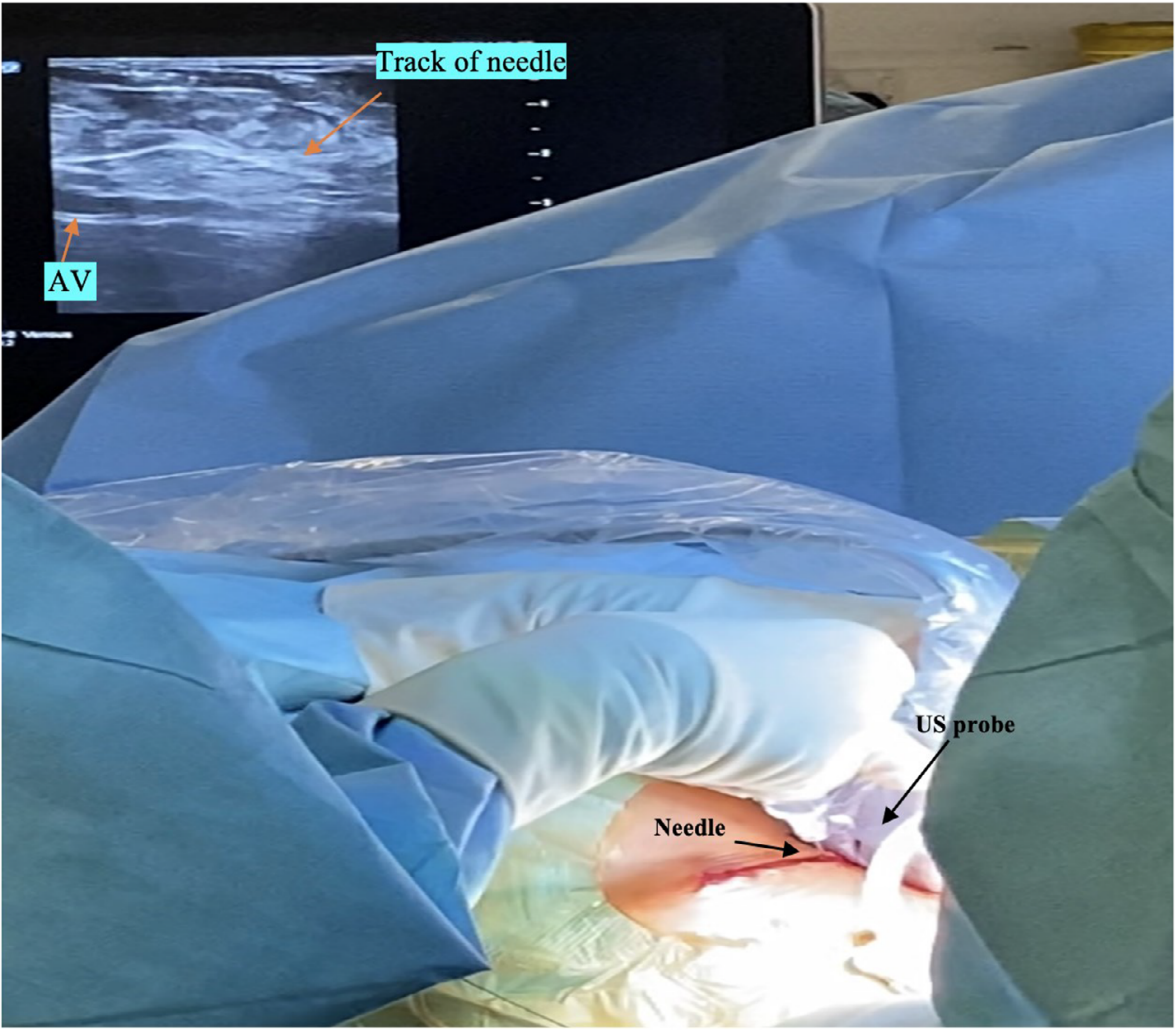
Demonstrating the position of the US probe and the needle for the US‐guided axillary venous (AV) puncture at the superior end of incision. [Colour figure can be viewed at wileyonlinelibrary.com]

With the probe in situ, 1% lidocaine was infiltrated from below the midpoint of the probe inferiorly and parallel to the deltopectoral groove. An incision was then made through the dermis only. An 18‐gauge needle was attached to a 10 mL syringe and advanced in short movements under slight negative pressure. This was tracked under US guidance until the needle tip pierced the vessel wall (Figure 3). The needle was confirmed as within the lumen by US and by venous backflow into the syringe. A 0.035‐inch J‐tipped guide wire was then advanced into the vessel after removing the syringe. A separate puncture was performed for each lead implanted.

**FIGURE 4:**
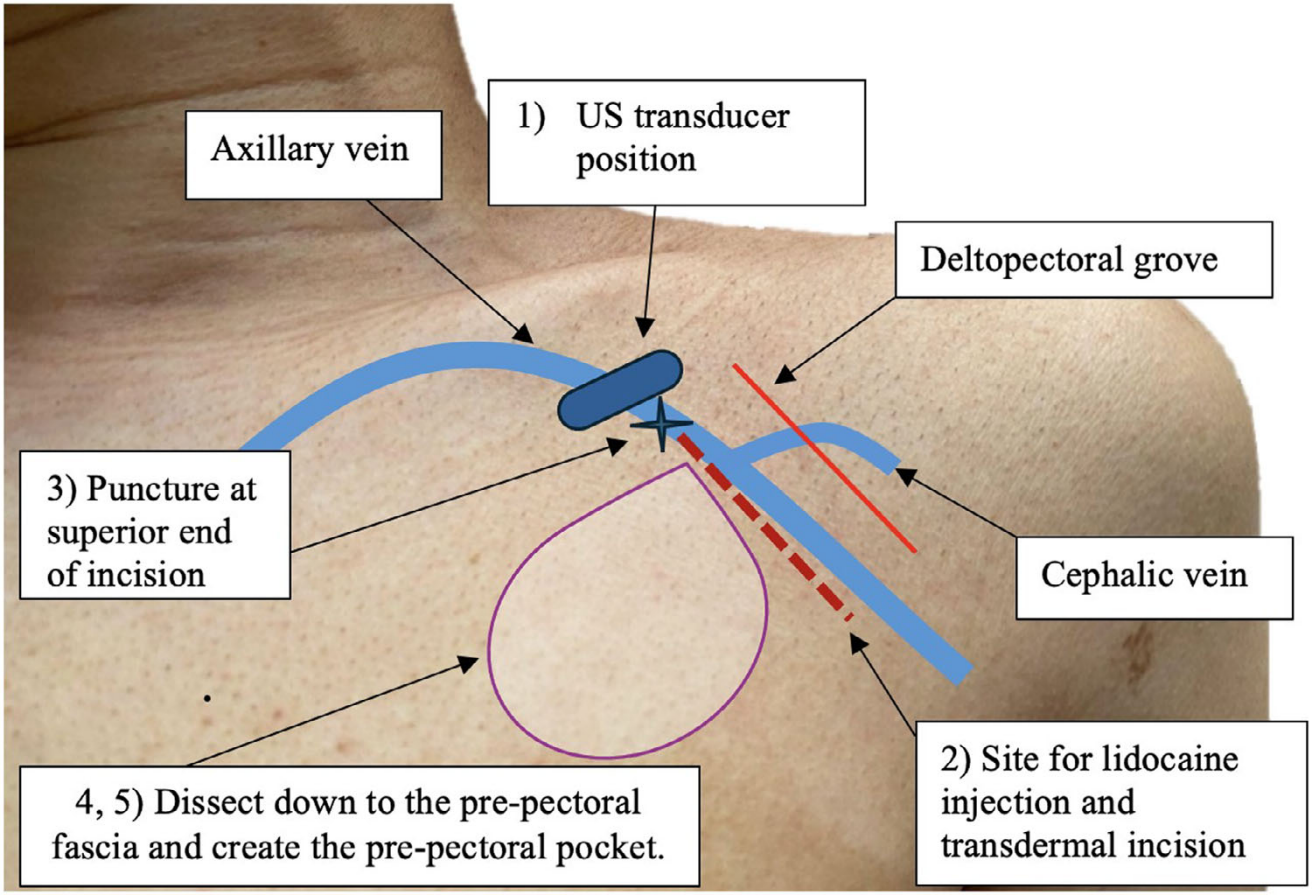
Illustration of procedural steps: **Step 1**: position the ultrasound transducer in the deltopectoral groove and move medially and upward toward the clavicle until the axillary vein and artery can be seen in the out‐of‐plane projection, with the vein in the middle of the imaging field. **Step 2**: inject the lidocaine and make a transdermal incision from below the midpoint of the probe inferiorly and parallel to the deltopectoral groove. **Step 3**: puncture at superior end of incision. **Step 4**: dissect down to pre‐pectoral fascia. **Step 5**: create prepectoral pocket. [Colour figure can be veiwed at wileyonlinelibrary.com] [Colour figure can be viewed at wileyonlinelibrary.com]

Cutting diathermy (Valleylab Force FX‐8C, Medtronic, Boulder, CO, USA) was used to dissect through the subcutaneous layer down to the pre‐pectoral fascia. Care was taken to free the J‐tipped guide wires from the subcutaneous fat. The medial edge of the incision was then lifted, and a pre‐pectoral pocket fashioned in the usual position (Figure 4).

After venous access, the remainder of the implant followed conventional practice. Post‐procedural care involved routine chest radiograph and a device check for pacing‐dependent patients. Patients were typically discharged on the same day.

### 2.3 Endpoints

Complications were recorded up to discharge. Efficacy was measured by procedural success and access time (needle to wire time). For comparison, access time was also measured in 21 control patients undergoing conventional fluoroscopy landmark‐guided access, performed by seven experienced operators. Radiation dose saving was estimated from measurement of fluoroscopy time for access, cumulative skin dose, and effective dose area product in controls. Strict institutional radiation protocols were used to minimize radiation exposure, including low frame rates (0.5–3 frames/s) [14], and proper collimation with other standard measures to keep exposure as low as reasonably achievable (ALARA protocol).

### 2.4 Statistics

Data were analyzed in R (R Foundation, Vienna, Austria) using RStudio Server version 1.0.153 (RStudio Inc., Boston, Massachusetts). All continuous variables are expressed as mean±SD, median (interquartile range) for skewed, and percentages for categorical data. Between‐group comparisons were performed using the Mann‐Whitney *U* test. A conversion factor of 0.2 was used to convert effective dose area product (Gy.cm^2^) into milliSieverts [15]. For contextualization, radiation doses were compared to exposure from chest radiography (0.02 mSv), and precision of the estimate was expressed as a 95% confidence interval (CI) [16].

## 3 Results

### 3.1 Baseline Characteristics

One hundred forty‐seven US‐guided axillary vein punctures were performed in 74 patients (age 72 ± 16 years, 58% male). Venous access was required for implantation of one (*n* = 6, 8%), two (*n* = 52, 71%), or three (*n* = 13, 17%) leads, or device upgrades (*n* = 3, 4%). For comparison, fluoroscopy‐guided access time and radiation exposure were measured in 21 patients undergoing implantation of one (*n* = 5), two (*n* = 9), or three (*n* = 7) leads by different operators.

### 3.2 Safety and Efficacy

There were no peri‐procedural complications relating to venous access, including pneumothorax. US‐guided axillary vein punctures were successful in 72 (97%) patients attempted. In the two patients with unsuccessful US‐guided access, the axillary vein was either not visualized (small caliber on subsequent venography) or situated deeply with prohibitively steep wire angulation.

### 3.3 Access Time and Learning Curve

**FIGURE 5:**
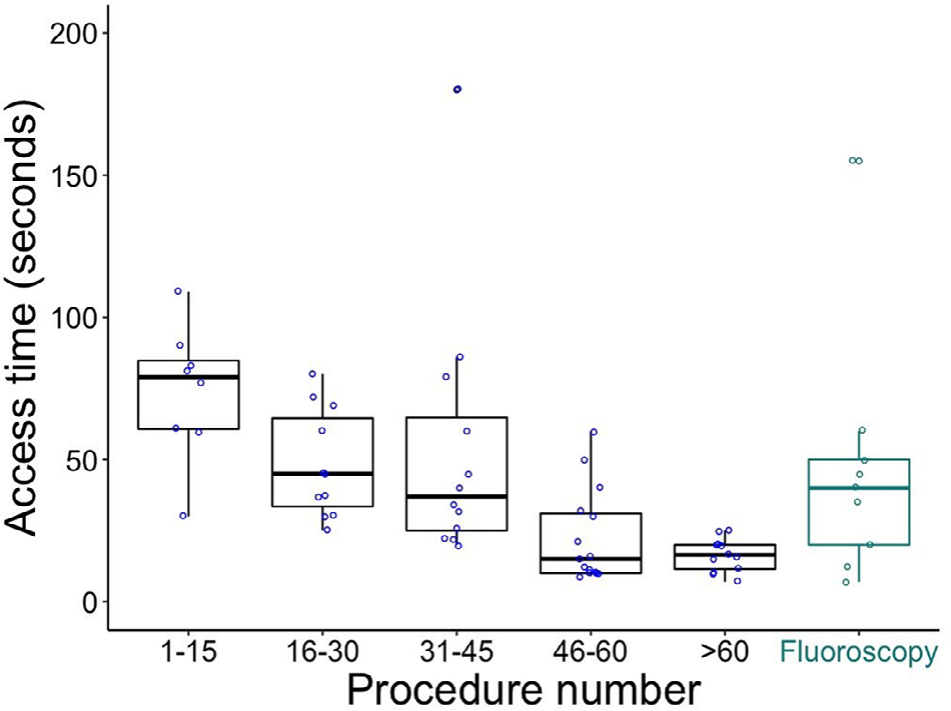
Access times for first puncture per patient decrease with experience. Statistically similar fluoroscopy‐guided access times for controls are shown in green for reference. One procedure requiring 506 s is not represented on the graph. [Colour figure can be viewed at wileyonlinelibrary.com]

US‐guided access time for all punctures was median 25 seconds (interquartile range [IQR]: 18,48), and ranged from 5 to 506 seconds per puncture. The longest procedure time (sixth procedure) required 506 seconds for the first puncture. One patient with low body mass index (BMI)and a collapsing vessel despite intravenous fluids needed comparatively longer time for access (180 seconds). All other access times were less than 120 seconds. Time for US‐guided access decreased from 81 (IQR: 61,90) to 17 seconds (IQR: 12,20) from the first to the last fifteen procedures (*p* < 0.001). Access times stabilized after approximately 45 procedures (Figure 5). First US‐guided puncture per patient was 30 seconds (IQR: 17,60) and was similar to first fluoroscopy‐guided puncture time (43 seconds, IQR: 24,58; *p* = 0.45).

### 3.4 Radiation Exposure

Sixty‐nine (96%) patients did not require fluoroscopy during US‐guided access. Three (4%) patients required 1 second fluoroscopy time after successful US‐guided access to confirm J wire position due to difficult passage.

Controls required 30 seconds (IQR: 15,78) of fluoroscopy time for access, resulting in 0.4 (IQR: 0.26,1.7) mGy cumulative skin dose, 0.09 (IQR: 0.03,0.17) Gy.cm2 effective dose area product, and equivalent to 1.3 (95% CI: 0.26,1.45) chest radiograph radiation exposure.

## 4 Discussion

This study placed a strong emphasis on the learning curve associated with US‐guided axillary venous access for cardiac device implantation, distinguishing it from previous studies that primarily focused on safety, efficacy, and technical feasibility [17, 18, 19]. Access time reduced from 81 seconds in the early cases to 16 seconds as experience increased and the learning curve stabilized after 45 procedures. This suggests that new operators will require less than 50 punctures to achieve stability and efficiency in clinical workflows. For cardiac electrophysiologists, adoption of the technique appears to have a high procedural success rate (97%) with no access‐related complications. Additional benefit is of radiation saving, equivalent to 1.3 chest radiographs [9, 14]. Although the study was a single‐center observational analysis with procedures performed by a single operator, this design allowed for a controlled and consistent assessment of skill acquisition over time.

In the previous randomized clinical trials, venous puncture is typically performed prior to skin incision using a standard vascular US probe. Although this approach preserves image quality, it can complicate dissection around the guidewire and may require modifications to the standard procedural workflow [6, 7, 9]. Alternatively, some studies reported the use of intrapocket US‐guided puncture following pocket creation, which often requires a small‐footprint or dedicated US probe which are not routinely available in standard practice [20, 21]. In our study, the skin incision was made inferior to the midpoint of the US probe and directed parallel to the deltopectoral groove, aligning laterally with the anatomical course of the axillary vein. This approach preserved the intact skin beneath the probe, allowing the use of US probes of varying sizes while maintaining optimal skin contact, minimizing air artifact, and enhancing image quality. The intact skin also facilitated smooth probe manipulation when required. Additionally, this technique allowed for pre‐puncture pocket creation without disrupting the US field, thereby supporting seamless integration into standard procedural workflows—particularly for operators who favor early pocket formation. The lateral incision orientation provided a more intuitive and controlled needle trajectory. A small volume of normal saline (<500 mL) was administered intravenously on the ipsilateral side to facilitate visualization of the axillary vein, and no adverse events related to fluid overlaid were observed. However, it should be performed with caution, guided by clinical judgement, particularly in patients at risk of volume overload such as those with heart failure.

US‐guided access was fast (approximately 30 seconds) and similar to fluoroscopy landmark‐guided access. Access time in other studies ranged between 2 and 7 minutes, including either local anesthetic administration or draping the probe [10, 13, 22]. This may be due to the position of the incision and prior operator experience of US‐guided femoral venous and arterial access.

In this study, we also noted that US‐guided venous access was successfully performed in patients with high BMI with no technical difficulty encountered. The BMI values ranged from 18.3 to 44.2, with a mean of 26±5.4. This observation aligns with existing literature suggesting that US guidance is particularly advantageous in patients with elevated BMI, due to improved imaging contrast from increased subcutaneous tissue [23, 24].

Operators strive to maintain radiation exposure as low as reasonably achievable (ALARA) [25]. The use of US‐guidance redefines what is reasonable during cardiac device implantation by obviating the need for fluoroscopy during venous access. A minority of patients did require brief fluoroscopy to confirm wire position where there was difficult passage into the right atrium. Previous studies have measured average procedural radiation usage, meaning that it is not possible to estimate the specific saving related to access. In this study, actual radiation exposure for standard fluoroscopy‐guided venous access was measured for a more precise estimate. This provided an estimated radiation saving comparable to one chest radiograph for the patient (plus additional exposure to the operator). This is a conservative estimate compared to other institutions, which have reported fluoroscopy times for venous access approximately six times longer [9, 14].

### 4.1 Study Limitations

This was a single‐center observational study with procedures performed by a single operator, ensuring consistency in assessing the learning curve despite limiting generalizability. The inclusion of 21 control cases performed by different operators using conventional methods provided a comparative framework to estimate radiation exposure for venous access, but it will vary according to center and operator. Modifications reported may limit US probe orientation within the pocket, but were feasible for the probes used in this study. Time for probe draping was not measured, although it was not a major obstacle in a previous report [13], and may be counterbalanced by time saved elsewhere. Follow‐up was limited to the time of discharge. Estimates for radiation dose saving were from a range of cardiac devices, but a limited number of patients.

## 5 Conclusion

US‐guided axillary venous access for cardiac device implantation is a feasible alternative to fluoroscopy‐guided access. Data presented quantify radiation savings and demonstrate a short learning curve for safe use with a high success rate.

## Data Availability

The data that support the findings of this study are available from the corresponding author upon reasonable request and will be provided exclusively for peer review purposes.
